# The environmental fate of polybrominated diphenyl ethers (PBDEs) in western Taiwan and coastal waters: evaluation with a fugacity-based model

**DOI:** 10.1007/s11356-016-6428-4

**Published:** 2016-03-29

**Authors:** Kieran O’Driscoll, Jill Robinson, Wen-Son Chiang, Yang-Yih Chen, Ruey-Chy Kao, Rory Doherty

**Affiliations:** School of Planning Architecture and Civil Engineering, The Queen’s University of Belfast, Northern Ireland, BT9 5AG UK; Tainan Hydraulics Laboratory, National Cheng Kung University, Tainan, Taiwan

**Keywords:** Persistent organic pollutants (POPs), Polybrominated diphenyl ethers, Fugacity modeling, Industrial emissions, Taiwan, Coastal waters

## Abstract

The environmental fate of polybrominated diphenyl ethers (PBDEs), a group of flame retardants that are considered to be persistent organic pollutants (POPs), around the Zhuoshui River and Changhua County regions of Taiwan was assessed. An investigation into emissions, partitioning, and fate of selected PBDEs was conducted based on the equilibrium constant (EQC) fugacity model developed at Trent University, Canada. Emissions for congeners PBDE 47, PBDE 99, and PBDE 209 to air (4.9–92 × 10^−3^ kg/h), soil (0.91–17.4 × 10^−3^ kg/h), and water (0.21–4.04 × 10^−3^ kg/h), were estimated by modifying previous models on PBDE emission rates by considering both industrial and domestic rates. It was found that fugacity modeling can give a reasonable estimation of the behavior, partitioning, and concentrations of PBDE congeners in and around Taiwan. Results indicate that PBDE congeners have a high affinity for partitioning into sediments then soils. As congener number decreases, the PBDEs then partition more readily into air. As the degree of bromination increases, congeners more readily partition to sediments. Sediments may then act as a long-term source of PBDEs which can be released back into the water column due to resuspension during storm events.

**Electronic supplementary material:**

The online version of this article (doi:10.1007/s11356-016-6428-4) contains supplementary material, which is available to authorized users.

## Introduction

Persistent organic pollutants (POPs) are toxic, recalcitrant chemicals which can bioaccumulate and biomagnify over long time periods within ecosystems (Jones and de Voogt 1999). Due to their persistence, POPs can have the potential to be transported over long distances to previously pristine regions distant to their point of origin (Bennett et al. 2001; ter Schure et al. 2004a, b). They can cause numerous health effects in humans, such as endocrine disruption and impairment of the immune system but with some being deemed potentially cancer causing (Darnerud 2003). Due to these and other health concerns, the Stockholm Convention on Persistent Organic Pollutants was implemented to protect human health and the environment from these chemicals. Since this is a global problem, and given the long range capabilities of these chemicals where any particular government or jurisdiction can protect its citizens from POPs, the Stockholm Convention is a global treaty, adopted in 2001 before entering into force in 2004. Because of the Stockholm Convention, member countries are legally obliged to provide POP inventories. Although implementation has improved estimates of POP inventories, they are still incomplete. POP inventories are calculated as Annual Release_POP_ = ∑Source strength × Activity where source strength is given as mass per input or output (Fiedler 2007). Some source categories are missing, particularly where data is not readily available, including, e.g., time series of emissions, and are still highly uncertain when available. Concerning POP sources, they are often manufactured for a specific purpose, e.g., for use as flame retardants or pesticides, or they can be created as byproducts of industrial processes such as combustion.

Polybrominated diphenyl ethers (PBDEs) are a group of POPs used as flame retardants. These chemicals are effective in the prevention of fire by their addition to everyday objects such as textiles, furniture, and electrical and electronic appliances (Besis and Samara 2012). The fire prevention process in PBDEs is dependent on the bromine atoms sequestering OH and H radicals that would promote flammable gas phase chain reactions during combustion (Rahman et al. 2001). As flame retardants, PBDEs have been manufactured to specifically have a long half-life in their host objects in order to prevent combustion. PBDEs were first manufactured in the 1970s and were generally marketed in three products: deca-BDE, penta-BDE, and octa-BDE formulations. These products all contain mixtures of different BDE chemical compounds, for example, the main congener existing in deca-BDE is BDE-209 (USEPA 2008). Deca-BDE is proposed by Norway as a candidate of POPs within the Stockholm Convention. The attachment F of deca-BDE was passed in the 11th Examination Committee about persistent organic pollutant on Oct. of 2015, and deca-BDE was suggested adding to the attachment A of convention. Thus, the elimination and reduction of deca-BDE and its pollution control may become an urgent issue to be resolved. The production of octa-BDE and penta-BDE was stopped completely by manufacturers in 2004 following growing health and environmental concerns (USEPA 2008). In 2009, commercial octa-BDE and penta-BDE were considered so hazardous that they became globally recognized as persistent organic pollutants with the potential of causing widespread harm, so much so that they were added to the list of POPs under the Stockholm Convention (UNEP 2009). Since items containing PBDEs are used more or less worldwide, it can be inferred that most people and ecosystems are at risk of some level of exposure. The most toxic of the PBDEs are the lower congeners. In comparison to commercial deca-BDE, they are more likely to accumulate due to their lower molecular weight (McDonald 2002). The debromination of larger congeners can produce more toxic daughter congeners (Schenker et al. 2008). BDE-209 has a higher molecular mass and does not bioaccumulate as effectively as some of the lower congeners, so lower concentrations will exist in the lipid cells of organisms for this congener, but BDE-209 has the capacity to break down into more toxic and easily absorbed congeners such as those existing in penta- and octa-BDE (McDonald 2002).

Health issues known to be associated with PBDE congeners include problems of the thyroid gland. Furthermore, exposure affects the liver and kidney structure with tumors developing in liver tissue (Besis and Samara 2012). Immune suppression has also been indicated as a possible health concern (U.S Department of Health and Human Services 2004). More recently, it has been suggested that PBDEs are endocrine disruptors, with women who have been exposed experiencing disruption to the menstrual cycle and increased difficulty with conception (Besis and Samara 2012).

### Sources of PBDEs

With the production and use of PBDEs gradually decreasing, it could be assumed that risks to ecosystem services and humans from exposure to PBDE congeners are also decreasing. However, even with decreasing production, we must also consider the life cycle of PBDE congeners beyond just their production. PBDE congeners are recalcitrant and potentially mobile between air, water, and soil phases and post production, they still can contaminate industrial sites and have a presence in industrial and domestic products that are still in use. Post usefulness, these products can still be a potential source of emission either as they are disposed in landfill or through attempts at recycling, i.e., disassembly of waste electronic and electrical products and remanufacture of PBDE containing plastics (Li et al. 2013). While there is a wealth on observation of the amounts of PBDEs in the domestic and indoor environments, e.g., Harrad et al. (2008), Webster et al. (2009), there are less detailed observations and models on the regional effects of PBDE mobility and their partitioning in soils, air, waters, and sediments. Despite all the negative publicity surrounding the PBDEs, Taiwan is not considered to be as stringent concerning policies on PBDEs as some other parts of the world, with the phase out of their use in electronics scheduled for 2016 (Taiwan 2014).

### Emissions to air

PBDEs are a group of semivolatile organic compounds (SVOCs) that are known to partition between the gas and particulate phases in the atmosphere. Partitioning is dependent on several properties including molecular weight, temperature, vapor pressure, and octanol-water coefficient. There are many pathways, or sources, of release of PBDEs to air, including combustion of fuels in power plants, emissions from E-waste facilities (Muenhor et al. 2010), and combustion of waste (Wyrzykowska-Ceradini et al. 2011). A Taiwanese study (Wang et al. 2011) found that combustion from industrial and domestic sources is a substantial emitter of PBDEs. The outcome of the study also indicated that emissions from power plants and exhausts could be one to three orders of magnitude higher than the PBDE content of indoor air, with indoor air dominated by lighter congeners. PBDEs have been shown to partition strongly to very fine particles which can account for long-range transport and incidence in remote regions (ter Schure et al. 2004a, b; Wang et al. 2007).

### Emissions to land

The disposal of materials treated with PBDEs, for example if disposed of in landfill sites or illegally dumped, permits PBDEs to enter the environment and ecosystems. Studies have shown that PBDE concentration levels are elevated in sewage treatment works (De Boer et al. 2003; Hale et al. 2002) and landfill sites (Besis and Samara 2012). Recycled or reused items will also no doubt harbor residual PBDEs, so, despite bans on production and use of PBDEs in items, they could still be indirectly incorporated in everyday objects containing recycled plastics or electrics (USEPA 2008). Fifty to eighty percent of recycled electrical and electronic waste is from countries with higher labor costs and is being exported to countries in East Asia (Besis and Samara 2012). This results in emissions from uncontrolled waste practices around electrical and electronic equipment forming a significant global source of PBDEs (Bi et al. 2007; Wong et al. 2007).

### Emissions to water

Elevated levels of PBDEs have been noted in particulate matter in sewage treatment works (De Boer et al. 2003) which are often discharged directly to controlled waters without tertiary treatment specifically designed to deal with POPs.

Taiwan is an example of a country where years of heavy manufacturing and production are likely to have resulted in anthropogenic environmental effects. Taiwan’s industry sector is a high economic contributor; however, following years of heavy manufacturing and production, it is now considered an area at risk from its industrial legacy. Taiwan’s manufacturing industry rapidly grew in the 1930s when many industrial plants were formed on the island by the Japanese (Rubinstein 2006). Taiwan’s industrial sector is still a high economic contributor and has moved to a high level of electronics and information technology production—particularly semiconductors. It also produces automobile and other vehicle parts, plastics, chemicals, and textiles (German Trade Office Taipei 2014). A substantial number of these will be treated with flame retardants, and it is likely, due to the present lack of precautionary legislation, that PBDEs in particular are still being stored and used in Taiwan and Taiwanese products. The Taiwanese Environmental Protection Agency has recently indicated that PBDEs are to be phased out in their electronic industry in 2016 (Taiwan 2014). So it can be assumed that PBDEs are still being used and in circulation, making the island a source of PBDE contamination, which may result in risks to its own inhabitants, ecosystems, and neighboring countries.

### Environmental modeling

Fugacity, or partial pressure, is generally used to define a compound’s tendency to “escape” or partition into other phases or compartments, such as soil, air, or water (Mackay and Paterson 1981). It is calculated as a function of the pressure of the gas in ideal conditions. Environmental compartments (e.g., soil, gas, water, sediment) that are in equilibrium will have equal fugacity values. Each compartment will also have a fugacity capacity, Z (mol/m^3^Pa), that considers concentration, temperature, and physicochemical properties, and expresses the affinity of a chemical for a particular compartment. This concept uses the assumption that the concentration of a compound (C) within a compartment is equal to the fugacity (f) multiplied by that compartment’s fugacity coefficient (Z). A compartment will have the highest chemical concentration where the Z value is highest. If the compartments are not in equilibrium, the difference in fugacities gives some indication of how close to equilibrium the system is and how high the concentration of a chemical in a particular phase is relative to other phases (Mackay et al. 2009). This has an advantage over the use of partition coefficients only, because if the system as a whole is not in equilibrium, then the equilibrium driving force can be quantified by the difference in fugacity between the two phases. Although fugacity modeling is not a substitute for detailed fate and transport modeling, it can provide valuable information that will inform monitoring and sampling programs that form the basis for numerical modeling approaches.

Here, we use the updated equilibrium criteria (EQC) model (Hughes et al. 2012) to consider the likely fate of PBDEs on a regional scale. The model has three tiers or levels. Level I considers the equilibrium distribution or steady state in a closed environment and suggests the phases or environmental compartments that a chemical will partition to. Level I output identifies the compartment where the fugacity capacity, Z, and chemical concentrations are likely to be highest. Levels II and III build on previous tiers by adding advective fluxes, degradation, and transport between compartments, eventually allowing residence times to be calculated. The EQC model was first described by Mackey et al. (1996) who applied it to a polyaromatic hydrocarbon (PAH), pyrene, and lead. The model has since been applied to many different scenarios for POPs and other chemicals. For example, to represent European PBDE emissions, Palm et al. (2002) developed a conceptual PBDE emissions model for Denmark. This model is used as a framework for our study and is discussed at length below. Beyer et al. (2000) used the EQC model to assess the long-range transport (LRT) potential of 18 POPs, including PCBs and organochlorine pesticides (OCPs). They showed that the EQC and other box models can be used to estimate characteristic travel distances of POPs while demonstrating a simple relationship between this distance and persistence. Using the RAIDAR model (Arnot et al. 2006), which is similar to the EQC model but also incorporates biota, Webster and Mackay (2007) estimated the fate and distribution of dioxins and furans generated in association with mining, oil, and gas camps in Northern Canada and found that they enter the local food web from soil, water, and vegetation, following initial deposition on the surrounding landscape.

The potential effects of PBDEs on the environment and on human health would suggest that their presence in Taiwan should be closely monitored. While this can be (and has been) done via regional sampling, this is expensive in comparison to the use of modeling software and is a lot more difficult to accurately complete, requiring more time and manpower (Mackay et al. 2009). It is however possible for the use of modeling predictions to be used in conjunction with sampling and monitoring methods to optimize costs and determine the environmental fate potential of the chemical in question.

This study was undertaken to develop a knowledge and understanding of PBDEs and their interactions and fate in the environment and in Taiwan in particular at present. A fugacity-based model, which seeks to imitate the partitioning and fate of the chemicals, was implemented for three of the most commonly occurring and potentially dangerous PBDE chemicals—BDE-47, BDE-99, and BDE-209. These representative congeners were also chosen to cover a variety of molecular weights that provide a range on likely mobility and fugacity across phases. These model realizations used physical-chemical properties for the different congeners and conditions relating to the Taiwan environment such as temperature and soil quality, which were researched and developed upon throughout the study.

## Materials and methods

### Conceptual model and domain

**Fig. 1:**
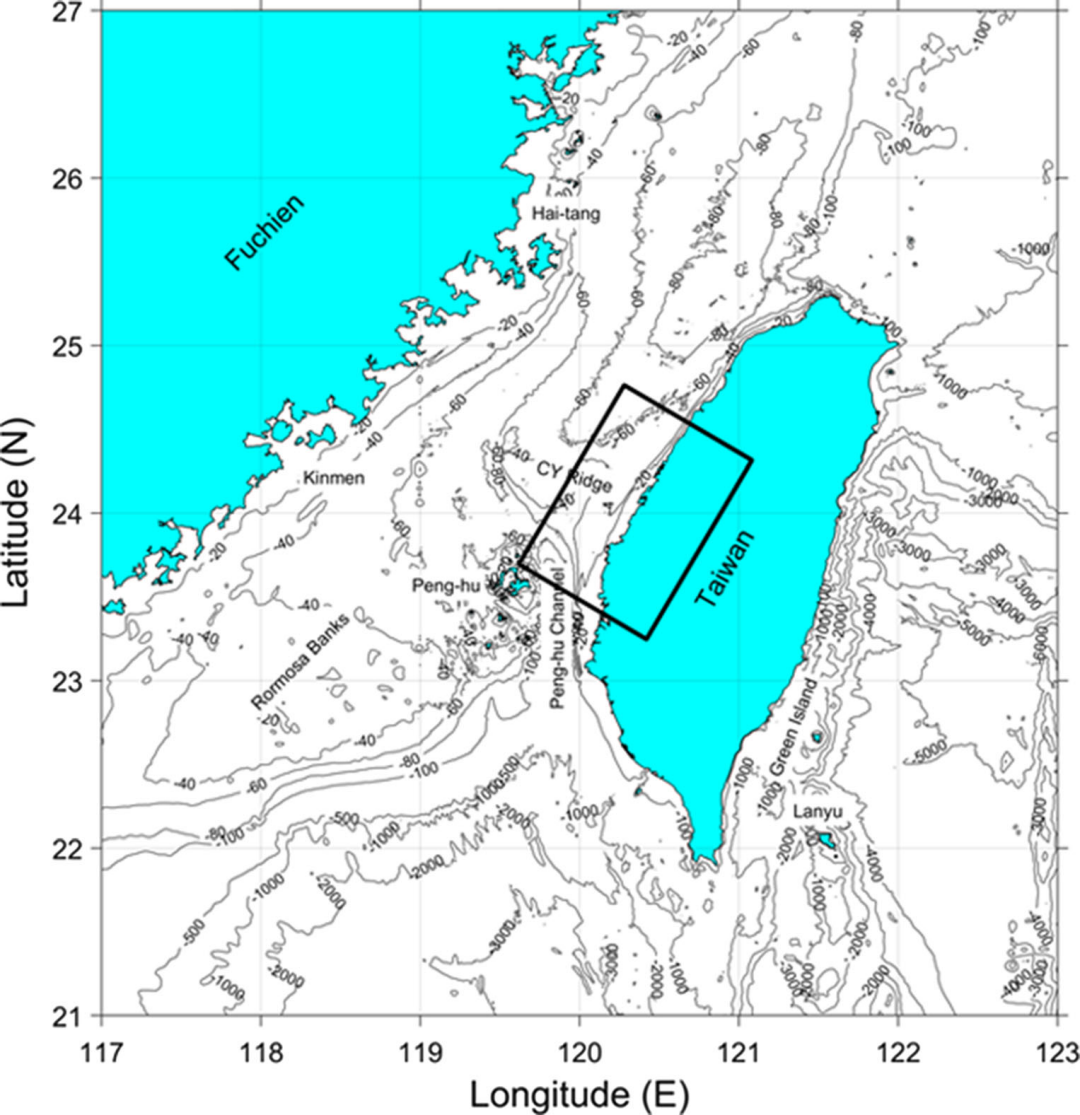
Model domain (*black box*) covers an area of 100 km × 100 km with 50 % of the domain on land and the remainder in the Taiwan Strait

The model domain chosen is a 100 km × 100-km area (10,000 km^2^) in western and coastal Taiwan (Fig. 1).It consists of the southwestern area of Changhua County as well as the northwestern area of Yunlin County which features the most built-up area in the proximity of the Zhuoshui River, and thereby, some of the highest users and emitters of PBDEs. The domain was configured such that 50 % of the model was on land and 50 % in the coastal area of the Taiwan Strait. On the ocean side, the model domain contains the northern end of the Peng-hu Channel, most of the CY Ridge, and extends northward beyond the CY Ridge. This domain was selected so that areas of interest on land (southwest Changhua County, northwest Yunlin County, and Zhuoshui River) were included, but also so that a reasonable adjacent area of the Taiwan Strait was included. This allows for deposition from both the Zhuoshui River and atmospheric deposition into the Taiwan Strait. This is particularly important given the dominant northeasterly to easterly Monsoon winds across Taiwan which will carry POPs emitted into air on the land side of the model domain into the Taiwan Strait side of the model domain (Lee and Chao 2003; Liang et al. 2000). Model depths of air, water, soil, and sediment compartments were chosen as 1000, 35, 0.2, and 0.05 m, respectively. Water depth was chosen based on the average water depth in the model domain. Values of compartment areas, depths, and volumes used in the model are given in Table 1.

**Table 1:** Values of compartment areas, depths, and volumes entered into the EQC model (Hughes et al. 2012)

Compartment Dimensions	Area (m^2^)	Depth (m)	Volume (m^3^)
Air	1.00E+10	1000	1.00E+13
Water	5.00E+09	35	1.75E+11
Soil	5.00E+09	0.2	1.00E+09
Sediment	5.00E+09	0.05	2.50E+08

The water depth of 20 m is constant and does not make allowances for variances in the depth of water close to the shoreline. Other assumptions include the depth of soils and sediments in the conceptual model area; they have been assumed to represent the depths of 0.05 and 0.2 m, respectively, as given in the EQC model input, which is considered reasonable.

The model results from this can be used to give a good representation of the presence of PBDEs across the western area of the island, because this is the most industrial and most inhabited area. The island is small enough so that northern and southern temperature differences do not impact on partitioning, and since PBDE production began after the island became industrialized, it is not expected that there will be a large difference in background levels across this area.

### Physical-chemical properties for model input

The EQC model requires the input of the physical-chemical properties of each of the PBDEs to determine how they will behave. Generally, PBDEs have low vapor pressure and solubility, which makes measuring their physical-chemical properties rather difficult (Palm 2001). Some of the physical-chemical information was relatively straightforward to obtain and has little scope for error; for example, molar mass and melting points have been well documented with similar results arising each time. Other properties, however, such as the solubility, vapor pressure, and partition coefficients, have more variance in their recorded values. Physicochemical parameters for the PBDE congeners used here are supplied in Supplementary Information Tables 1–9.

### Soil and sediment properties for model input

Properties of soil and sediment relating to the model domain are required for the determination of the solid-water partition coefficient, K_oc_. These properties are the fraction organic carbon (F_oc_) values for the regional soils and sediments. They are important for determining the PBDE’s affinity to solids, as a higher organic carbon content gives soils and sediments a higher capacity for PBDE partitioning due to the absorbent nature of carbon. The fraction organic carbon (F_oc_) content for soil was obtained using an average value of soil organic carbon measurements taken in the Changhua region of Taiwan which was calculated to be 0.0121 (Tsui et al. 2013). The F_oc_ for sediment was calculated to be 0.00752 from the conversion of the average of 30 values taken along the southwest coast of Taiwan (Kao et al. 2006). This value is lower than the soil F_oc_, which is considered reasonable since sediment generally will consist of less porous sands and silts.

### Estimation of PBDE emissions from Taiwan

The input of estimated emission rates of the model domain into the EQC model is also required. This has a large effect on the output given in two ways: first, the concentrations of the chemicals in the environment will be higher or lower according to the emissions input, and second, model output should vary greatly depending on the compartment to which the chemicals have been emitted. Obtaining a reasonable estimate for emissions is therefore highly important but is a complex process with much ambiguity. Sources can be split into additive, industrial, and degradation sources. Additive sources refer to the PBDEs which have been emitted from the host objects in which they were incorporated. From here, they enter the atmosphere and groundwater via dust and waste disposal leachate. Considering the wide range of items which are treated with flame retardants, it can be assumed that all occupied buildings are a source of PBDEs. Industrial sources however refer to sources originating from industry in particular. These can be split into two groups: wastage from manufacturing plants that incorporate PBDEs into their products and combustion emissions from industrial plants generally. Given that octa- and penta-BDE commercial products are no longer in manufacture (USEPA 2008), it could be assumed that wastage from manufacturing plants will produce mostly emissions of BDE-209 into the air and effluent leaving the plant. In the case of combustion sources such as power plants, waste incinerators, and mineral processing, the highest concentrations being emitted into the atmosphere were of the higher brominated congeners such as BDE-209, but relatively high concentrations of the lower BDE-47 and BDE-99 congeners were also found. Finally, degradation sources have been identified as noteworthy sources of the lower brominated congeners such as BDE-47 and BDE-99, where degradation of the larger compounds such as BDE-209 can sometimes create these smaller congeners in their host environment. The receptors of these emissions with regard to the EQC model are air, water, soil, and sediment in Taiwan.

Palm et al. (2002) developed a conceptual PBDE emissions model for Denmark that is representative of European PBDE emissions. The model estimates the emissions from households per capita and forms a good starting point for the estimation of PBDE emissions in this study. In this particular case, there are a number of modifications that are required to fit with our specific conceptual model and fugacity model domain. The Danish emissions model considers household emissions only, but due to the presence and legacy of industry and waste disposal in Taiwan, there is a requirement to consider PBDE emissions from industrial combustion, production, and wastage. There should also be some allowance made for the degradation of BDE-209 as a direct contribution to BDE-47 and BDE-99 emission levels. The total emission estimates are also based on the population of Denmark. In this case, emissions therefore had to be tailored to suit the population in Taiwan. This assumed that emissions per household are the same for both areas, which is likely to be the case as consumption of goods such as household objects are unlikely to change a great deal between the two countries. Adaptation for industrial emissions involved scaling the emissions of all three congeners up by a factor of 3 to take into account industrial and waste disposal sources. This number was chosen to maintain a conservative approach. It also involved scaling the emissions of BDE-47 and BDE-99 up by factors of 1.02 and 1.13 respectively to take into account the breakdown of BDE-209. These figures came from the fact that 2 and 13 % of BDE-47 and BDE-99 congeners are considered to have come from the degradation of BDE-209 (Schenker et al. 2008). The second change involved converting the total emissions into that of the model domain. This was allowed for by dividing the total brominated flame retardants by a figure of 5,357,000, the population of Denmark, to convert the emissions into a “per person” format. The figure obtained was then multiplied by 650 × 9000, which is the average residents per square kilometers (German Trade Office Taipei 2014) multiplied by the land area of the model domain.

All other methodology in the flow chart calculation was kept consistent with that used by Palm et al. (2002). PBDE emission rates for Taiwan incorporating domestic emissions were calculated based on a study of PBDE emission rates in Denmark and are summarized in Table 2.

**Table 2:** Emission rates for each of the three congeners into the various compartments

Compartment	Emissions (kilograms per hour × 10^−3^)		
	BDE-47	BDE-99	BDE-209
Air	4.90	7.12	92.5
Water	0.212	0.309	4.04
Soil	0.914	1.34	17.4
Total	6.026	8.769	113.94
Total emissions per annum (kg)	52.79	76.82	998.1

Emission rates are greatest for BDE-209: they are an order of magnitude greater than either of BDE-47 and BDE-99, which are both emitted at similar rates. The three congeners are emitted mostly in air, about five times more than soil emissions and about a factor of four greater than water emissions. There are no PBDE emissions directly to sediments. Emission ratios are the same for all three congeners: 81 % to air, 15 % to soil, and 4 % to water.

### Temperature data from Taiwan

The temperature of the environment is also an important factor to consider when modeling POPs as it can change the output data greatly. Temperature is important because it has an effect on variables such as concentrations in the gas phase and the speed at which chemicals degrade or transform (Dalla Valle et al. 2007). The results of temperature change are well illustrated in Supplementary Information (Table 10) where air sampling in different regions showed that in comparison to spring, much higher concentrations of the PBDEs (higher brominated congeners in particular) were measured in the autumn season (Lin 2012).

In the modeling conducted, however, it is more relevant to consider ground rather than air temperatures. This is because the PBDEs will almost automatically partition to soil and sediment so the soil temperature will provide a more accurate result. It should however be noted that a source of error usually exists by doing this, where the PBDEs being emitted into the atmosphere will be of a higher temperature than those partitioning into the soil. In Taiwan however, the average soil temperature, obtained from Lu et al. (2008) is in fact 22 °C—the same as that of the average air temperature, so this error is not present to the same extent. Lu et al. (2008) also identified that soil temperatures can change over the year by as much as 10 °C.

Temperature changes are accounted for using enthalpy of phase change values. An enthalpy in a compartment at a constant pressure is equal to the amount of heat used or emitted by the molecules, which plays a big part in the phase changes and partitioning of the chemicals.

Temperature adjustments were also applied to the degradation half-lives of the congeners in their various compartments since temperature change can have a large effect on half-lives. This is done using the activation energy values for the congeners which quantify the minimum amount of inner energy required by the chemical in question to undergo reaction. Activation energies used on PBDEs in the past have been 10,000 J/mol for half-life in air and 30,000 J/mol for the half-lives in water, soil, and sediment (Palm Cousins 2012). Since no congener-specific values are available, it can be assumed that these are rough estimates only. Degradation slows under lower temperatures (Dalla Valle et al. 2007), so it is expected that half-lives will increase in these model calculations.

### Previously measured concentrations

Concentrations measured previously in Taiwan and Asia are being used as a comparison between measured samples and the model output. These measured concentrations form the basis of model comparison with observations. It has been assumed that readings from the western area of Taiwan will be broadly consistent with those expected of the conceptual model since the climate will not have changed much. The water compartment has not been considered because limited data was available, and sampling data from the Hong Kong region of China mostly found that PBDE values were below the detection limit, which can be attributed to the hydrophobic nature of the PBDEs.

## Results and discussion

### Model output

**Fig. 2:**
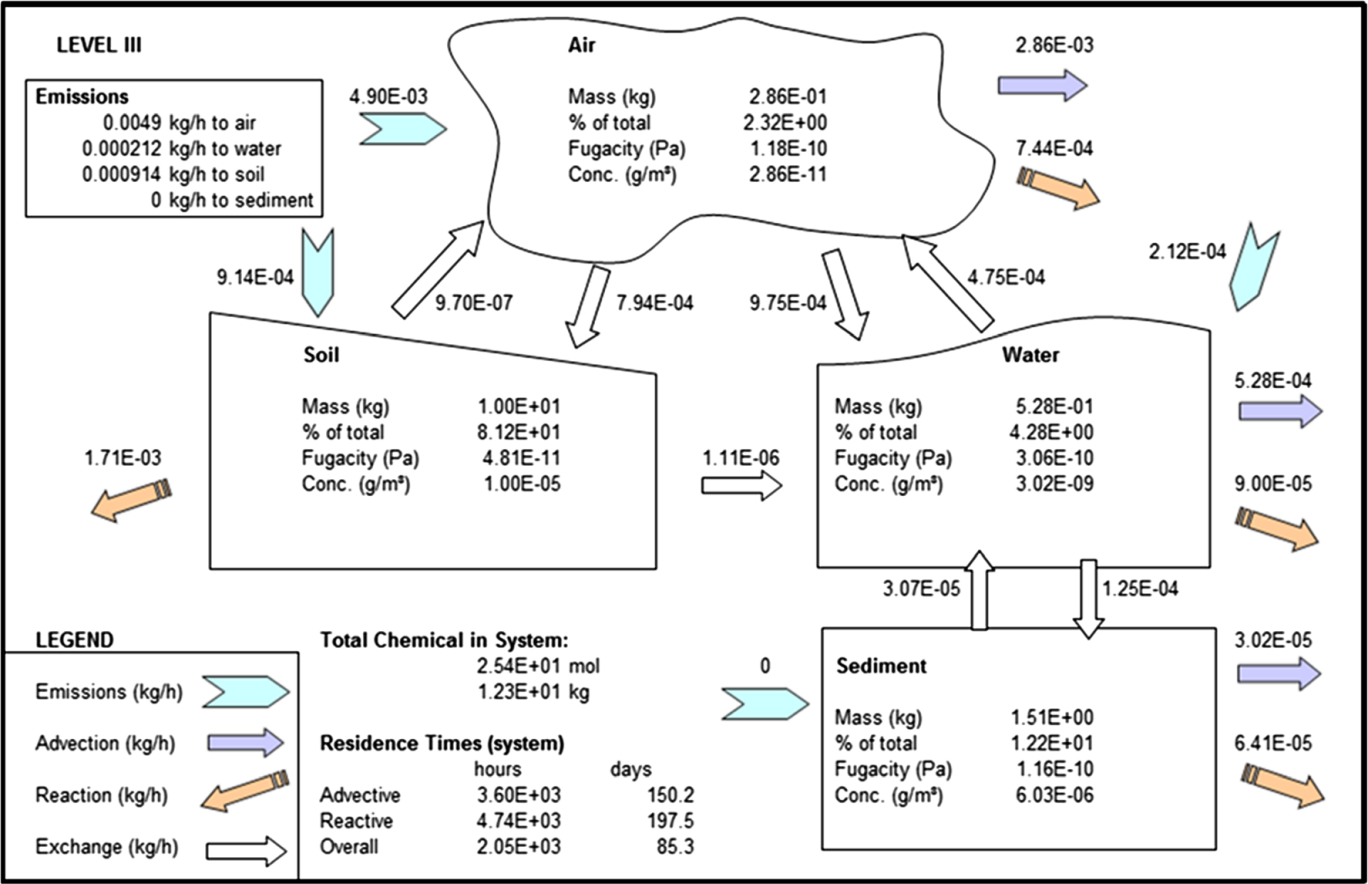
EQC model output for BDE-47

**Fig. 3:**
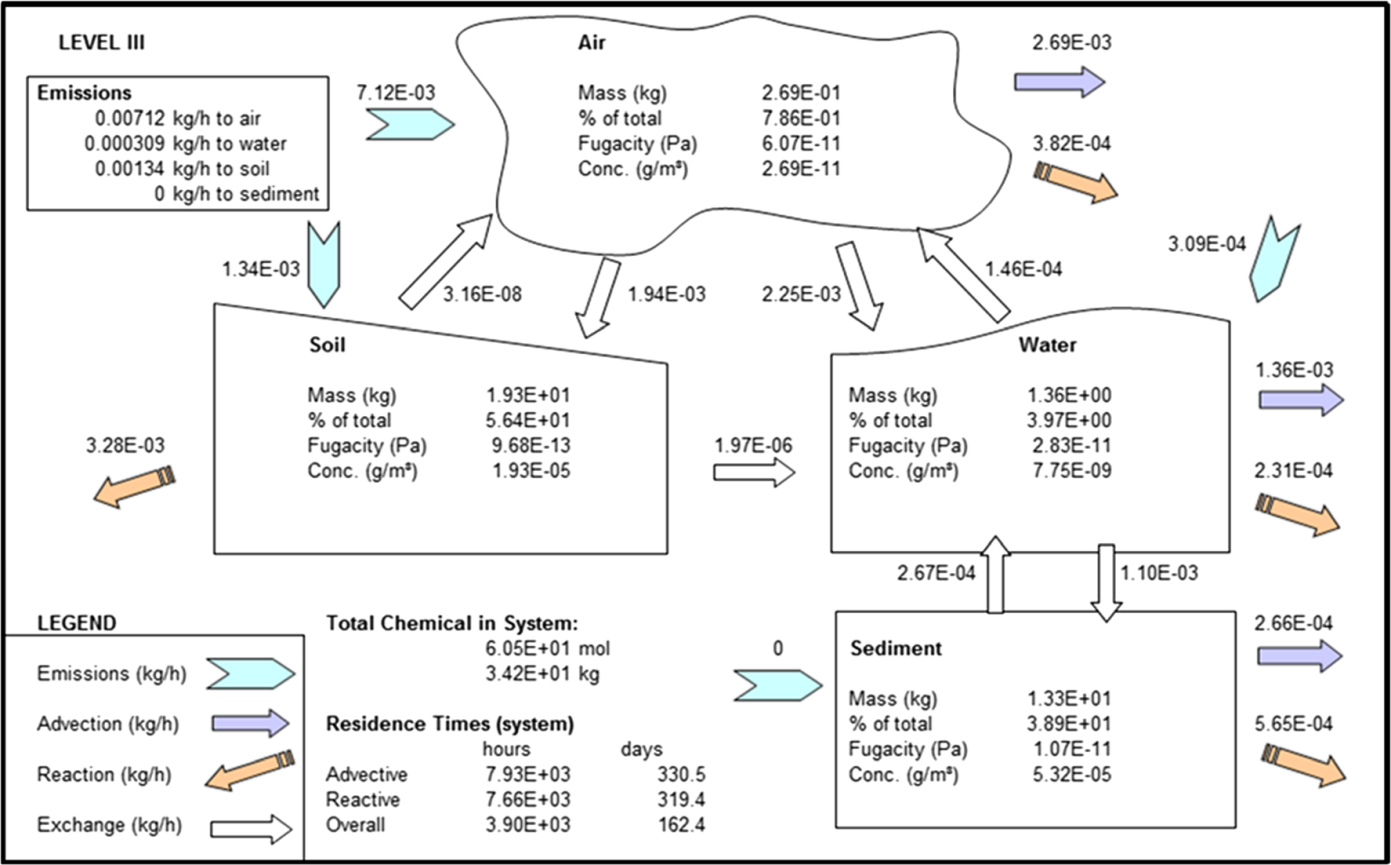
EQC model output for BDE-99

**Fig. 4:**
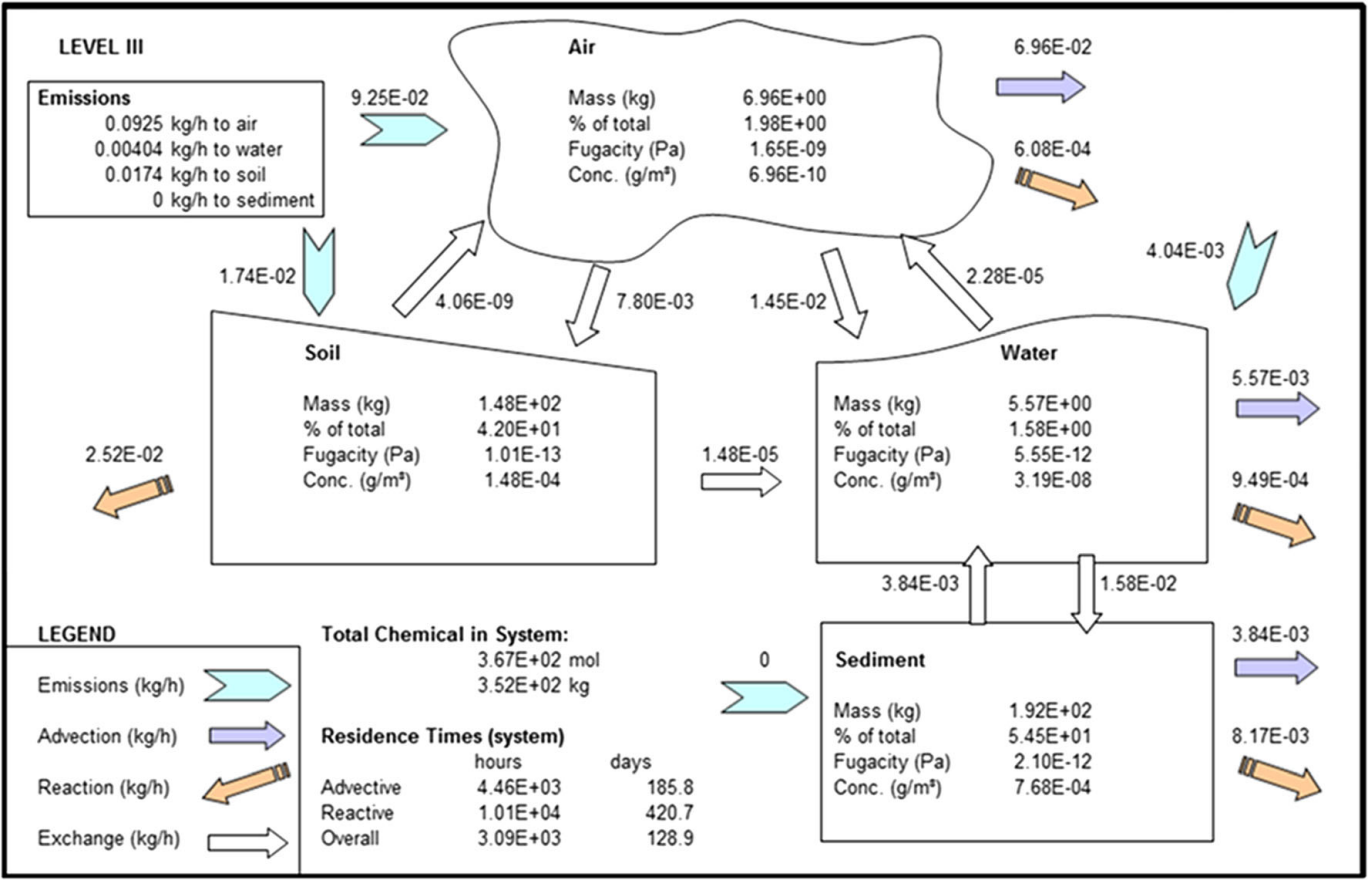
EQC model output for BDE-209

We note here that the model results are given for steady state conditions. Simple model mass balances show that fluxes into the model domain are approximately equal to those exiting the model domain (Figs. 2, 3, 4, Table 3), and the model code is written so the model is automatically spun up until a steady state has been achieved. Calculating the difference between fluxes into and out of the model domain and dividing the total mass in the system by the amount of BDE being added to the system every hour shows that the system has taken between 10 and 40 years to reach this state. This is a reasonable result since PBDEs have been manufactured since the 1970s (U.S Department of Health and Human Services 2004).

**Table 3:** Final distributions of the congeners in each of the four compartments.

Congener						
Compartment	BDE-47		BDE-99		BDE-209	
	Mass [kg]	Percent	Mass [kg]	Percent	Mass [kg]	Percent
Air	0.286	2.32	0.269	0.786	6.96	1.98
Water	0.528	4.28	1.36	3.97	5.57	1.58
Soil	10.0	81.2	19.3	56.4	148	42.0
Sediment	1.51	12.2	13.3	38.9	192	54.5
Total	12.3	100	34.2	100	352	100

### BDE-47

Most of this POP entering the model domain by emissions to air is lost: 58 % is advected out of the domain, 20 % to the water compartment, approximately 16 % to the soil compartment, and approximately 15 % is degraded (Fig. 2). However, there is some returning to the air compartment by volatilization from water and diffusion from soil compartments which maintains the mass balance and keeps the system in steady state. Of the 20 % transmitted from the air compartment to the water compartment, half is returned to air through volatilization. About 13 % of BDE-47 entering the water compartment ends up in the sediment compartment, with 25 % returned from sediment to water. Of the 16 % moving to the soil compartment from the air compartment, very little is returned to air, while less than 2 % is advected to the water compartment from soils.

The net result of all of this exchange is that the soil compartment becomes the greatest repository for BDE-47, with 81 % (10 kg) of the total; next, 12 % (1.51 kg) is in the sediment compartment, with 4 and 2 % in water and air compartments, respectively.

### BDE-99

Almost all of the emissions to the air compartment are lost: 38 % is lost by advection out of the system, 32 % is lost to water, while 27 % is lost to soil. Of the total lost to water, 6 % is returned to air by volatilization and a very small fraction is revolatilized from soil (Fig. 3).

Eighty-six percent of the POP entering water is from air-sea exchange, with 14 % entering directly through water, and a very small amount is advected from soil. Forty-three percent of the total amount entering water is transferred to sediment, reflecting the hydrophobicity of the compound, while 24 % is returned to the water column due to diffusion and resuspension.

The net result is that of the total of the BDE-99 remaining within the model domain, 56.4 % (19.3 kg) is contained in soil, 38.9 % (13.3 kg) in sediment, 4 % (1.36 kg) in water, and less than 1 % in air.

### BDE-209

Most of the POP emitted to the air compartment is lost. It is seen that advection by air out of the model domain is the major sink out of the system (75 %, Fig. 4). A substantial amount (16 %) of the POP in air enters the water column, most of which sorbs to organic material because of the hydrophobicity and lipophilicity of the POP, and will end up in the sediment. In fact, almost all of the chemical entering water ends up in the sediment, with a small amount being advected out of the system, and a small portion being volatilized back to air. There is also a substantial amount, 24 %, returned from sediment to water which will be due to diffusion processes, including resuspension; however, most of this settles back down into sediment. The amount of BDE-209 deposited on soil is approximately half of that deposited in water. Very little of the POP deposited on soil is revolatilized back to the atmosphere.

Although deposition rates to soil are greater than to sediment, the fact that the half-life of BDE-209 in sediment is four times greater than in soil (Wania and Dugani 2003) means that sediment ends up being the greatest repository for the congener remaining in the system. The net result of all these processes is that 54.5 % (192 kg) is contained in the sediment compartment, 42 % (148 kg) in soil, 1.6 % (5.57 kg) in water, and 2 % (6.96 kg) in air.

### Ratios in compartments

Distributions of the different congeners in each of the four compartments are summarized in Table 3. Most of the chemical remaining in the model domain is found in either soil or sediment. For the lightest of the three congeners, BDE-47, most of the chemical sorbs to soil (81 %), but this proportion decreases as we move from lighter to heavier weight congeners. The pattern is repeated in water: proportion of the congener contained in the compartment decreases with weight of the congener (higher numbered congeners are heavier). The opposite outcome is found to be the case in sediment: the proportion of the total increases with increasing congener number (weight). This can be explained by the different partitioning coefficients of the congeners: air-water partitioning coefficients (K_AW_), octanol-air partitioning coefficients (K_OA_), and octanol-water partitioning coefficients (K_OW_) (Table 4). Since the soil-air partitioning coefficient is high for all three congeners, there is a relatively large flux from air to soil in all three cases. The return flux, from soil to air, is much smaller, by factors of approx. 10^3^, 10^5^, and 10^6^, for the cases of BDE-47, BDE-99, and BDE-209, respectively.

**Table 4:** Partitioning coefficients of the three PBDE congeners (log_10_).

Congener			
Partitioning coefficient (log_10_)	BDE-47	BDE-99	BDE-209
Air-water K_AW_	−1.7	−2.9	−1.9
Octanol-water K_OW_	6.6	7.28	9.97
Soil-air K_OA_	8.3	10.2	11.8

For the case of the octanol-water partitioning coefficient, K_OW_, all three congeners are hydrophobic. However, BDE-209 is more hydrophobic than BDE-99 which is more hydrophobic than BDE-47. Since half of the BDE-47 entering water is returned to air, it becomes available for partitioning to soil. Because BDE-99 is more hydrophobic than BDE-47, less than a tenth of that entering water is returned to air, thus a lesser portion is available for partitioning in soil. Finally, because of its very high hydrophobicity, almost all the BDE-209 entering water is not available for return to air (only 2 parts in 1000), and this accounts for the very high portion in sediment.

### Model validation

The model was validated by comparing results (model output) with available concentration measurements in the various compartments and is shown in Table 5. As highlighted in Table 2 and the section on model output, emissions to air make up the majority of the inputs for all three congeners, followed by emissions to soil then water. With all three congeners, there is a significant loss to advection out of the model with deposition to water and soil removing the majority of the pollutant mass in air. Model concentrations in air are in reasonably good agreement with available measured values. A sensitivity analysis of input parameters and corresponding outputs is given in the Supplementary Information (Table 10). For both BDE-47 and BDE-99, model concentrations are a factor of 2–3 higher than the highest values measured by Li et al. (2011) in the South and East China Sea regions. For BDE-209, model results are within the range of values measured by Li et al. (2011) for the region around the city of Tainan.

**Table 5:** Comparison of model results with available concentration measurements

Compartment	Concentrations		
	BDE-47	BDE-99	BDE-209
Air (pg/m^3^)			
Modeled	28.6	26.9	69.6
Sampled—Taiwan			Tainan^b^
Range	0.41–12.7^a^	0.15–11.3^a^	75 ± 16.8–18 ± 1.3
Water (pg/L)			
Modeled	3	7.77	31.83
Sampled—Taiwan	No data	No data	No data
Soil (ng/g dw)			
Modeled	7	13	99
Sampled—Taiwan	75–104^c^	41–84^d^	260–330^e^
Sediment (ng/g dw)			
Modeled	4	35	512
Sampled—Taiwan	<0.5 × 10^−4^–2.7^f^	<0.5 × 10^−4^–2.2^f^	0.3–44.6^f^

^a^Lin et al. (2012), based on South and East China Sea atmospheric sampling

^b^Lin et al. (2012)

^c^WHO (1994)—sourced from Palm et al. (2002) and based on readings at an industrial area. Value for all Tetra-BDE congeners

^d^WHO (1994)—as above. Value for all penta-BDE congeners

^e^WHO (1994)—as above for BDE-209

^f^Li et al. (2011)—sampling from sediment in the East China Sea

In soil, model results are somewhat less than the measured values: for BDE-47, they are a factor of 10 less; for BDE-99, a factor of 3 or more less; and for BDE-209, a factor of 3 less.

In sediment, model results are at the high end of measured values: for BDE-47, they are a factor of 2 greater than the highest measured values and for BDE-99 and BDE-209, a factor of 10 higher than the measured values. The authors have been unable to find any published data for PDBE in coastal waters around Taiwan. This would suggest that monitoring of this media is of upmost importance. Although model results are generally higher in air and sediment and lower in soil relative to measurements, they are not too unreasonable given the limitations of the model.

There are a number of reasons for model inconsistencies with measured values: emission rates could be unrepresentative. This is a possibility due to the lack of sound emission data present in literature, and it is possible that the emission ratio between soil, air, and water is incorrect. This applies particularly to BDE-209—since it has a lower vapor pressure, it could be expected that the percentage of emissions to air would be lower and emissions to water and soil would be higher.

Half-lives used in this study for soils, sediments, and water did not change with congener, and half-lives for soil and water were chosen to be the same. This indicates that these values were an estimate, thus there is a high likelihood of introducing error to the model. A higher half-life in soil would increase the burden in that compartment.

It should be considered that the soil sample data given may not be truly representative. The measured values in Table 5 (refs. c, d, e) refer to measurements of various congeners with the same degree of bromination as the congeners used in the study, as opposed to single BDE-47 and BDE-99 congeners. These measurements are taken from an industrial area in Taiwan so soil would likely have higher concentrations—particularly if this area has high flame retardant usage. Furthermore, the samples could have been taken during the winter period in Taiwan, which would cause an increase in the half lives and partitioning in the soil and therefore elevated concentrations. Finally, these measurements date from 1994, that is, before the risks of PBDE usage were fully identified, which opens up the possibility that they may have been used more frequently at the time of sampling.

This discrepancy also occurred in a previous study by Palm et al. (2002). They too identified that the high discrepancy could be due to the reasons listed above. Based on the fact that emissions in this study will have changed from the study of Palm et al. (2002), it is most likely that the discrepancy was caused by the half-life data being incorrect or the soil concentrations being unrepresentative—or both of these.

Tables 3 and 5 suggest that sediments then soils are the predominant sink for PBDEs and this is in agreement with similar work on the fate and transport of PBDEs (Environment Canada 2006). The sediment sink for PBDEs in this case is also helped by the proximity of source areas to the Taiwan Strait; in this case, the model domain areas for soil and water (sediment) compartments as outlined in the “Materials and methods” section is equal. This allows emissions to air to directly impact on soil and water with PBDEs not remaining in the water column but travelling through to sediments. Ideally, to determine the source of error, more soil and sediment samples should be obtained. This would disclose how representative the soil sample results above are and therefore if actions need to be taken to increase the accuracy of the model.

## Conclusions

This study has shown that the use of fugacity modeling can give a reasonable indication of the behavior, partitioning, and concentrations of PBDE congeners in and around Taiwan and reinforces the assumption that they are largely present in the Zhuoshui River area of Taiwan, where a substantial part of Taiwanese industry is based. The model results indicate that PBDE congeners generally have a high affinity for partitioning in soils and sediments and that all congeners will to some extent, in equilibrium conditions, partition in the soils compartment where they become relatively immobile. This tendency decreases with decreasing congener number, with the results suggesting that BDE-47 will more readily partition in air, with the capability of some long-range transport when partitioning in air—which is a risk factor for areas beyond Taiwan. BDE-209 has been found to have the highest concentrations within the model domain environment, which was to be expected due to the lesser worldwide restriction on this congener. Given present estimated emission rates, however, the PBDEs are also found in the sediment compartment of the model domain due to their hydrophobicity, with increasing bromination leading to a higher percentage partitioning to sediments. They also have a high tendency for transfer from the sediment compartment to the water compartment. These sediments have the potential to be a future contaminant source area. For example, O’Driscoll et al. (2013) demonstrated that storm activity remobilizes POPs on sediment which leads to increased volatilization (O’Driscoll 2014). O’Driscoll et al. (2014) have also demonstrated that increasing intensity of storm events due to climate change will mobilize greater quantities of sediments and promote greater exchange of contaminants back into the water column. This is a significant risk because this is where the remobilized sediment PBDEs have the capacity to enter the food chain. The present model outputs indicate that there is also risk of long-range transport due to the PDBE emissions to air from the Zhuoshui River area—a risk that is present for all three congeners and not just due to the equilibrium partitioning effects of BDE-47 as stated above. This suggests that Taiwan’s PBDE problem will not only affect Taiwan but also areas outside the model domain, including coastal areas of Taiwan.

It is therefore of paramount importance that production and usage of PBDEs should be limited in Taiwan as soon as possible to avoid the creation of future source areas in sediments as well as long-range transport of PBDEs in air. Generally speaking, modeled concentrations were broadly consistent with available measurements. However, the PBDE concentrations showed a discrepancy between modeled results and sampled results in soils. The soil data used to validate the model comes from studies that try to assess point sources from industry rather than determine regional background concentrations (Doherty et al. 2015; McIlwaine et al. 2015, 2014). To rectify this, it is recommended that this fugacity modeling approach should be used to design a regional approach to obtain representative samples for soils, sediment air, and coastal waters. Investigations into the half-life measurement of the PBDE congeners across a variety of media would also be highly useful, since at the time of modeling, only estimates were available. Specific emission data from Taiwan is also a significant data gap at present so information on present usage in Taiwan and probable emission rates of industrialized countries would be highly beneficial. Other weather-related factors such as wind speed and direction and the effect of precipitation on concentrations are also a possibility in pursuing more detailed information, which could be used to build a larger picture of the issue of PBDE burden in Taiwan. This, coupled with specific hydrodynamic modeling of coastal waters, would allow a more quantitative overview of sources and sinks of PBDEs around Taiwan.

## Electronic supplementary material

Below is the link to the electronic supplementary material.

ESM 1 (DOCX 27 kb)

## References

[CR1] Arnot JA, Mackay D, Webster E, Southwood JM (2006). Screening level risk assessment model for chemical fate and effects in the environment. Environ Sci Technol.

[CR2] Bennett DH, Scheringer M, McKone TE, Hungerbühler K (2001). Predicting long-range transport: a systematic evaluation of two multimedia transport models. Environ Sci Technol.

[CR3] Besis A, Samara C (2012). Polybrominated diphenyl ethers (PBDEs) in the indoor and outdoor environments—a review on occurrence and human exposure. Environ Pollut.

[CR4] Beyer A, Mackay D, Matthies M, Wania F, Webster E (2000). Assessing long-range transport potential of persistent organic pollutants. Environ Sci Technol.

[CR5] Bi X, Thomas GO, Jones KC, Qu W, Sheng G, Martin FL, Fu J (2007). Exposure of electronics dismantling workers to polybrominated diphenyl ethers, polychlorinated biphenyls, and organochlorine pesticides in South China. Environ Sci Technol.

[CR6] Dalla Valle M, Codato E, Marcomini A (2007). Climate change influence on POPs distribution and fate: a case study. Chemosphere.

[CR7] Darnerud PO (2003). Toxic effects of brominated flame retardants in man and in wildlife. Environ Int.

[CR8] De Boer J, Wester PG, Van Der Horst A, Leonards PEG (2003). Polybrominated diphenyl ethers in influents, suspended particulate matter, sediments, sewage treatment plant and effluents and biota from the Netherlands. Environ Pollut.

[CR9] Doherty R, McIlwaine R, McAnallen L, Cox S, O’Sullivan G, Megson D (2015). Assessment of polycyclic aromatic hydrocarbons in an urban soil dataset. Environmental forensics : Proceedings of the 2014 I.E. Conference, Special Publications.

[CR10] Canada E (2006). Ecological screening assessment report on polybrominated diphenyl ethers (PBDEs), Environment Canada.

[CR11] Fiedler H (2007). Global POPs inventories. Organonhaline Compounds.

[CR12] German Trade Office Taipei (2014) German Trade Office, Taipei [WWW Document]. Taiwan Econ. Data

[CR13] Hale RC, La Guardia MJ, Harvey E, Matt Mainor T (2002). Potential role of fire retardant-treated polyurethane foam as a source of brominated diphenyl ethers to the US environment. Chemosphere.

[CR14] Harrad S, Ibarra C, Diamond M, Melymuk L, Robson M, Douwes J, Roosens J, Dirtu AC, Covaci A (2008) polybrominated diphenyl ethers in domestic indoor dust from Canada, New Zealand, United Kingdom and United States. Environ Int 34(2):232–238. doi:10.1016/j.envint.2007.08.008 10.1016/j.envint.2007.08.008 17897716

[CR15] Hughes L, Mackay D, Powell DE, Kim J (2012). An updated state of the science EQC model for evaluating chemical fate in the environment: application to D5 (decamethylcyclopentasiloxane). Chemosphere.

[CR16] Jones KC, de Voogt P (1999). Persistent organic pollutants (POPs): state of the science. Environ Pollut.

[CR17] Kao S-J, Shiah F-K, Wang C-H, Liu K-K (2006). Efficient trapping of organic carbon in sediments on the continental margin with high fluvial sediment input off southwestern Taiwan. Cont Shelf Res.

[CR18] Lee H-J, Chao S-U (2003) A climatological description of circulation in and around the East China Sea. Deep-Sea Res II Top Stud Oceanogr 50 (6–7):1065–1084. doi:10.1016/S0967-0645(03)00010-9

[CR19] Liang W-D, Jan JC, Yang TY (2000) Climatological wind and upper ocean heat content in the South China Sea. Acta Oceanographica Taiwanica 38:91–114

[CR20] Li X et al (2011) Evaluation of atmospheric sources of PCDD/Fs, PCBs and PBDEs around a steel industrial complex in northeast China using passive air samplers. Chemosphere 84(7):957–963 10.1016/j.chemosphere.2011.06.004 21726889

[CR21] Li Y, Li J, Wang L (2013). Recycling of PBDEs containing plastics from waste electrical and electronic equipment (WEEE): a review. 2013 I.E. 10th International Conference on E-Business Engineering.

[CR22] Lin L-F (2012). Atmospheric concentrations and dry deposition of polybrominated diphenyl ethers in Southern Taiwan. Aerosol Air Qual Res.

[CR23] Lu S, Hwang L, Tang K (2008). Soil temperature regimes in the Lienhuachih Area of Central Taiwan. Taiwan J For Sci.

[CR24] Mackay D, Arnot JA, Webster E (2009) The Evolution and Future of Environmental Fugacity Models. Ecotoxicol Model Emerg Topics Ecotoxicol; 2. doi:10.1007/978-1-4419-0197-2

[CR25] Mackay D, Paterson S (1981). Calculating fugacity. Environ Sci Technol.

[CR26] Mackay D, Di Guardo A, Paterson S, Cowan CE (1996). Evaluating the environmental fate of a variety of types of chemicals using the EQC model. Environ tox chem.

[CR27] McDonald TA (2002). A perspective on the potential health risks of PBDEs. Chemosphere.

[CR28] McIlwaine R, Cox SF, Doherty R (2015). When are total concentrations not total? Factors affecting geochemical analytical techniques for measuring element concentrations in soil. Environ Sci Pollut Res Int.

[CR29] McIlwaine R, Cox SF, Doherty R, Palmer S, Ofterdinger U, McKinley JM (2014) Comparison of methods used to calculate typical threshold values for potentially toxic elements in soil. Environ Geochem Health. doi:10.1007/s10653-014-9611-x 10.1007/s10653-014-9611-x 24760621

[CR30] Muenhor D, Harrad S, Ali N, Covaci A (2010). Brominated flame retardants (BFRs) in air and dust from electronic waste storage facilities in Thailand. Environ Int.

[CR31] O'Driscoll K, Mayer B, Ilyina T, Pohlmann T (2013). Modelling the cycling of persistent organic pollutants (POPs) in the North Sea system: fluxes, loading, seasonality, trends. J Mar Syst.

[CR32] O'Driscoll K (2014). Air-sea exchange of legacy POPs in the North Sea based on results of fate and transport, and shelf-sea hydrodynamic ocean models. Atmosphere.

[CR33] O’Driscoll K, Mayer B, Su J, Mathis M (2014). The effects of global climate change on the cycling and processes of persistent organic pollutants (POPs) in the North Sea. Ocean Sci.

[CR34] Palm A (2001). The environmental fate of polybrominated diphenyl ethers in the Centre of Stockholm— assessment using a multimedia fugacity model.

[CR35] Palm Cousins A (2012). Modelling the fate of organic contaminants in the Baltic Sea application of the POPCYCLING-Baltic model.

[CR36] Palm A, Cousins IT, Mackay D, Tysklind M, Metcalfe C, Alaee M (2002) Assessing the environmental fate of chemicals of emerging concern: a case study of the polybrominated diphenyl ethers. Environ Pollut 117(2):195–213. doi:10.1016/S0269-7491(01)00276-7 10.1016/s0269-7491(01)00276-7 11916035

[CR37] Rahman F, Langford KH, Scrimshaw MD, Lester JN (2001). Polybrominated diphenyl ether (PBDE) flame retardants. Sci Total Environ.

[CR38] Rubinstein MA (2006) Taiwan: a new history, expanded edition, M.E. Sharpe

[CR39] Schenker U, Soltermann F, Scheringer M, Hungerbühler K (2008). Modeling the environmental fate of polybrominated diphenyl ethers (PBDEs): the importance of photolysis for the formation of lighter PBDEs. Environ Sci Technol.

[CR40] Taiwan EPA (2014). Environmental policy monthly XV, 1–12.

[CR41] ter Schure AFH, Agrell C, Bokenstrand A, Sveder J, Larsson P, Zegers BN (2004). Polybrominated diphenyl ethers at a solid waste incineration plant II: atmospheric deposition. Atmos Environ.

[CR42] ter Schure AFH, Larsson P, Agrell C, Boon JP (2004). Atmospheric transport of polybrominated diphenyl ethers and polychlorinated biphenyls to the Baltic Sea. Environ Sci Technol.

[CR43] Tsui C, Guo H, Chen Z (2013). Estimation of soil carbon stock in Taiwan arable soils by using legacy database and digital soil mapping. Soil processes and current trends in quality assessment.

[CR44] UNEP (2009) Stockholm convention on persistent organic pollutants (POPs) press release: governments unite to step-up reduction on global DDT reliance and add nine new chemicals under international treaty. http://chm.pops.int/Convention/Media/Pressreleases/COP4Geneva9May2009

[CR45] U.S. Department of Health and Human Services (2004) Toxicological profile for polybrominated biphenyls and polybrominated diphenyl ethers

[CR46] USEPA (2008). An exposure assessment of polybrominated diphenyl ethers.

[CR47] Wang L-C, Lee W-J, Lee W-S, Chang-Chien G-P (2011). Polybrominated diphenyl ethers in various atmospheric environments of Taiwan: their levels, source identification and influence of combustion sources. Chemosphere.

[CR48] Wang Y, Jiang G, Lam PKS, Li A (2007). Polybrominated diphenyl ether in the East Asian environment: a critical review. Environ Int.

[CR49] Wania F, Dugani CB (2003) Assessing the long-range transport potential of polybrominated diphenyl ethers: a comparison of four multimedia models. Environmental Toxicology and Chemistry SETAC 22(6):1252–1261 12785581

[CR50] Webster E, Mackay D (2007) Modelling the environmental fate of dioxins and furans released to the atmosphere during incineration. CEMC Report 200701

[CR51] Webster TF et al (2009) Identifying transfer mechanisms and sources of decabromodiphenyl ether (BDE 209) in indoor environments using environmental forensic microscopy. Environ Sci Technol 43(9):3067–3072 10.1021/es803139w PMC2722073 19534115

[CR52] Wong MH, Wu SC, Deng WJ, Yu XZ, Luo Q (2007). Export of toxic chemicals—a review of the case of uncontrolled electronic-waste recycling. Environ Pollut.

[CR53] World Health Organization (1994) Environmental health criteria 162: brominated diphenyl ethers. Geneva, Switzerland

[CR54] Wyrzykowska-Ceradini B, Gullett BK, Tabor D, Touati A (2011). Waste combustion as a source of ambient air polybrominated diphenylethers (PBDEs). Atmos Environ.

