# Supplementary material for: The environmental fate of polybrominated diphenyl ethers (PBDEs) in western Taiwan and coastal waters: evaluation with a fugacity-based model
Source: Environ Sci Pollut Res Int. 2016 Mar 29;23:13222–34. doi: 10.1007/s11356-016-6428-4 (PMC4912977; doi:10.1007/s11356-016-6428-4)
Supplement: Supplementary file 1 — (DOCX 27 kb) [file 11356_2016_6428_MOESM1_ESM.docx]

**Supplementary Information**

Table 1 Solubility Values for BDE-47

| **Solubility Value (mg/l)** | **Source** | **Details** |
| --- | --- | --- |
| 0.001-0.002 | US EPA (2010) | Experimental |
| 1.46 x 10^-3^ | Palm et al (2002) | Experimental |
| 1.09 x 10^-2^ | Palm (2001) | Experimental |

Since the US EPA’s research into these properties is most recent, the midpoint of their range given is used for input into the model. This is consistent with Palm et al (2002)’s value, where the (2001) value is deemed an outlier since it is an order of magnitude larger than the other values.

Model input: **0.0015mg/l**

Table 2 Vapour Pressure Values for BDE-47

| **Vapour Pressure (Pa)** | **Source** | **Details** |
| --- | --- | --- |
| 2.5 x 10^-4^ | US EPA (2010) | Experimental |
| 1.86 x 10^-4^ | US EPA (2010) | Experimental |
| 2.15 x 10^-4^ | US EPA (2010) | Experimental |
| (2.6-3.27) x 10^-4^ | Palm (2001) | Experimental |
| 3.19 x 10^-4^ | Palm et al (2002) | Experimental |
| 3.21 x 10^-5^ | Palm (2001) | EPIWIN Estimate |

Since the US EPA’s research into these properties is most recent, the average of their values given is used for input into the model. This is broadly consistent with other values obtained from sampling; however the estimated value is an order of magnitude lower than this.

Model input: **2.17 x 10^-4^ Pa**

Table 3 Obtained Octanol-Water Partition Co-efficient Values for BDE-47

| **Log K_ow_** | **Source** | **Details** |
| --- | --- | --- |
| 6.01 – 6.77 | US EPA (2010) | Experimental |
| 6.81 | US EPA (2010) | Experimental |
| 5.87 – 6.16 | Palm (2001) | Experimental |

Again the average of the US EPA (2010) values was used for model input.

Model input: **6.60**

Table 4 Solubility Values for BDE-99

| **Solubility Value (mg/l)** | **Source** | **Details** |
| --- | --- | --- |
| 9 x 10^-7^ – 2.4 x 10^-3^ | US EPA (2010) | Experimental |
| 0.009 | US EPA (2010) | Experimental |
| 7.86 x 10^-5^ | Palm (2001) | EPIWIN Estimate |

The US EPA’s values were again used to determine the value for model input. The lower limit of 9 x 10^-7^ mg/l however was obtained in 1994 and is very low, so is therefore deemed an outlier. The average was therefore taken of 2.4 x 10^-3^ and 0.009.

Model input: **5.7 x 10^-3^ mg/l**

Table 5 Vapour Pressure Values for BDE-99

| **Vapour Pressure (Pa)** | **Source** | **Details** |
| --- | --- | --- |
| 6.82 x 10^-5^ | US EPA (2010) | Experimental |
| 1.76 x 10^-5^ | US EPA (2010) | Experimental |
| 3.63 x 10^-5^ | US EPA (2010) | Experimental |
| (2.9-7.3) x 10^-5^ | Palm (2001) | Experimental – Commercial Penta-BDE |
| 4.69 x 10^-5^ | Palm (2001) | Experimental – Commercial Penta-BDE |
| 3.25 x 10^-6^ | Palm (2001) | EPIWIN Estimate |

Since the US EPA’s values are experimental and specifically for the BDE-99 congener, the average of their values are used for model input. This is still broadly consistent with Penta-BDE values; however the estimated value is an order of magnitude lower than this.

Model input: **4.07 x 10^-5^ Pa**

Table 6 Obtained Octanol-Water Partition Co-efficient Values for BDE-99

| **Log K_ow_** | **Source** | **Details** |
| --- | --- | --- |
| 6.53 – 7.66 | US EPA (2010) | Experimental |
| 7.32 | US EPA (2010) | Experimental |
| 7.21 | US EPA (2010) | Experimental |
| 7.13 | US EPA (2010) | Experimental |
| 7.66 | US EPA (2010) | Experimental |
| 6.46 – 6.97 | Palm (2001) | Experimental |

Again the average of the US EPA (2010) values was used for model input.

Model input: **7.28**

Table 7 Solubility Values for BDE-209

| **Solubility Value (mg/l)** | **Source** | **Details** |
| --- | --- | --- |
| <0.001 | US EPA (2010) | Experimental – Commercial Deca-BDE |
| 2.5 x 10^-2^ | Palm (2001) | Experimental |
| 1 x 10^-4^ | Palm (2001) | Experimental – Commercial Deca-BDE |
| 2.8 x 10^-11^ | Palm (2001) | Estimate – Commercial Deca-BDE |
| 1.3 x 10^-8^ | Palm et al (2002) | Estimate |

In this case the values are more widespread and there is a lack of experimental data. However, since Deca-BDE is made up of 98% BDE-209 experimental data on Deca-BDE can be assumed broadly accurate. In comparison to BDE-47 and BDE-99’s values however 2.5 x 10^-2^ seems rather high, so the value of 1 x 10^-4^ was chosen for input. As illustrated with BDE-47 and BDE-99, estimated data tends to be lower than sampled data.

Model input: **1 x 10^-4^ mg/l**

Table 8 Vapour Pressure Values for BDE-209

| **Vapour Pressure (Pa)** | **Source** | **Details** |
| --- | --- | --- |
| 4.63 x 10^-6^ | US EPA (2010) | Experimental – commercial Deca-BDE |
| 1 x 10^-5^ | Palm (2001) | Experimental – commercial Deca-BDE |
| 5.8 x 10^-11^ | Palm (2001) | Estimate |
| 5.42 x 10^-11^ | Palm et al (2002) | Suspected estimate |

Making the same assumption as for the solubility where the estimated data is lower than reality, the experimental data for Commercial Deca-BDE was used. The US EPA’s value was implemented because the value from Palm (2001) again seemed rather high in comparison to the other congeners, which decreased by order of magnitude between the two congeners.

Model input: **4.63 x 10^-6^ Pa**

Table 9 Octanol-Water Partition Co-efficient Values for BDE-209

| **Log K_ow_** | **Source** | **Details** |
| --- | --- | --- |
| 6.27 | US EPA (2010) | Experimental – commercial deca-BDE |
| 11.15 | Palm et al (2002) | Suspected estimate |
| 9.97 | Palm (2001) | Experimental |
| 12.11 | Palm (2001) | Estimate |

The average of the experimental values given by Palm (2001) and US EPA (2010) was used for model input in this case. This is because the others are experimental values, and because the US EPA value was lower than the BDE-47 and BDE-99 values. This is not expected because BDE-209 has a lower vapour pressure, making it more hydrophobic.

Model input: **9.97**

Table 10 MODEL SENSITIVITY ANALYSIS

| **Input** | **Output Analysed** | **Input value** | **Output Value** | **Comments** |
| --- | --- | --- | --- | --- |
| Activation Energy (J/mol) | Half life in sediment (h) | 20,000  **30,000**  40,000 | 15,600  **16,300**  17,000 | Temperature kept constant.  Increasing half life with increasing activation energy. |
| Temperature (^o^C) | Half life in sediment (h) | 21  **22**  23 | 17,000  **16,300**  15,600 | Activation energy constant. Half life decreases in higher temperatures. |
| Fraction organic Carbon (FOC) (%) | Solid-water partition co-efficient. (soil) | 0.00652  **0.00752**  0.00852 | 10,390  **11,890**  13,470 | Organic carbon-water partition co-efficient kept constant.  Increases with FOC. |
| Enthalpy of phase change (J/mol) | Vapour Pressure (Pa) | 76,730  **86,730**  96,730 | 1.584 x 10^-4^  **1.521 x 10^-4^**  1.459 x 10^-4^ | Temperature kept constant at model value. Vapour pressure decreasing with increasing enthalpy. Results expected to be similar for solubility and octanol-water partition co-efficient. |
| Temperature (^o^C) | Vapour Pressure (Pa) | 21  **22**  23 | 1.348 x 10^-4^  **1.521 x 10^-4^**  1.713 x 10^-4^ | Enthalpy kept constant. Vapour pressure increasing with increasing temperature. |
| Temperature (^o^C) | Solubility (mg/l) | 21  **22**  23 | 1.342 x 10^-3^  **1.380 x 10^-3^**  1.419 x 10^-3^ | Enthalpy kept constant. Solubility increasing with increasing temperature. |
| Temperature (^o^C) | Octanol-water partition co-efficient (K_ow_) | 21  **22**  23 | 47,150,000  **45,190,000**  43,330,000 | Enthalpy kept constant. K_ow_ decreasing with increasing temperature. |
| Octanol-water partition co-efficient (logK_ow_) | Organic carbon-water co-efficient (K_oc_) | 6.555  **6.655**  6.755 | 1,256,000  **1,581,000**  1,991,000 | Seth et al method of calculation. Higher K_ow_ led to higher K_oc_ output. |
| Organic carbon-water co-efficient | Solid-water co-efficient | 1,571,000  **1,581,000**  1,591,000 | 11,810  **11,890**  11,960 | FOC kept constant. Solid-water co-efficient increases with K_oc._ |
| Vapour Pressure (Pa) | Henry’s Law Constant (Pa m^3^/mol) | 2.560 x 10^-5^  **2.560 x 10^-6^**  2.560 x 10^-7^ | 339.3  33.93  3.393 | Solubility kept constant. Higher vapour pressure increased Henry’s Law. Solubility would have opposite effect given equation  H = (P/S)x Mo (Equation 5.1) |

REFERENCES

Palm, A., 2001. The Environmental Fate of Polybrominated Diphenyl Ethers in the Centre of Stockholm - Assessment Using a Multimedia Fugacity Model. PhD diss., IVL.

Palm, A., Cousins, I.T., Mackay, D., Tysklind, M., Metcalfe, C., Alaee, M., 2002. Assessing the environmental fate of chemicals of emerging concern: a case study of the polybrominated diphenyl ethers. Environ. Pollut. 117, 195–213.

USEPA, 2010. An Exposure Assessment of Polybrominated Diphenyl Ethers. EPA/600/R- 08/086F, May 2010, pp.378.
